# Supplementary material for: External negative pressure transiently reduces intravenous pressure and augments the arteriovenous pressure gradient in the affected limb segment
Source: PLoS One. 2024 Dec 5;19(12):e0315231. doi: 10.1371/journal.pone.0315231 (PMC11620561; doi:10.1371/journal.pone.0315231)
Supplement: Attachment — Submitted filename: review PONE-D-24-09979 .docx [file pone.0315231.s001.docx]

This manuscript describes a study investigating the effect of an intermittent negative pressure treatment on the intravenous and intraarterial pressure in a cohort of volunteers. The authors concluded that the treatment resulted in a significant decrease in the intravenous pressure and therefore in an increase in the pressure gradient both in the supine and in the sitting position. The conclusion is well supported by the data. The study is well conducted, the methodology is sound, the statistical analysis has been performed appropriately and rigorously and the manuscript is overall well written. All data underlying the findings have been made fully available by the authors. Minor proofreading is needed:

Line 59 to 61: The authors wrote: “Although the systemic effects of LBNP are well documented (3), the mechanisms underlying the positive clinical effect of INP remain unclear, but may relate to oscillations in shear stress within the macro- (4) and micro-vasculature (5,6)” Since this is the first time the acronym “LBPN” appears in the text, you should spell it out.

Lines 286: The authors wrote: “Temporal changes during the during both postures are presented in Figure 1”. I think this should be “Temporal changes during both postures are presented in Figure 1”

Line 469 to 472: The authors wrote: “Similarly, the arterial pressure measurements we obtained were sensitive to changes in limb position, and mechanical perturbation (e.g. tapping or flushing) of the artery itself, therefore, we are therefore confident that our local arterial pressure measures would detect any relevant influence of an external pressure change” The word “therefore” is spelled twice. Please correct into “Similarly, the arterial pressure measurements we obtained were sensitive to changes in limb position, and mechanical perturbation (e.g. tapping or flushing) of the artery itself, therefore, we are confident that our local arterial pressure measures would detect any relevant influence of an external pressure change”
