# Supplementary material for: External negative pressure transiently reduces intravenous pressure and augments the arteriovenous pressure gradient in the affected limb segment
Source: PLoS One. 2024 Dec 5;19(12):e0315231. doi: 10.1371/journal.pone.0315231 (PMC11620561; doi:10.1371/journal.pone.0315231)
Supplement: Attachment — Submitted filename: Response to reviewers 1.docx [file pone.0315231.s002.docx]

**Response to reviewers 21-5-24**

***External negative pressure transiently reduces intravenous pressure and augments the arteriovenous pressure gradient in the affected limb segment.***

Many thanks for the kind comments and assistance with improving our manuscript. Responses to the individual points raised are below, with the relevant changes tracked within the updated manuscript.

**Reviewer 1**

*”Saphenous venous (n = 15), and dorsalis pedis artery pressure (n = 3) were recorded via pressure catheter, alongside beat-by-beat systemic cardiovascular parameters.“ Can you clarify the precise meaning of "N"? If "N" represents the number of different measurement points, then it seems that the measurement errors for arteriovenous pressure are not at the same level.¨*

Thank you for this request for clarification, these are indeed the number of observations obtained from our sample for saphenous intravenous pressure (*n* = 15), and intraarterial pressure from the dorsalis pedis artery (*n* = 3). We have added the further clarification that the arterial pressure measurements obtained from the beat-by-beat finger cuff measurements (Nexfin) were used for the estimation of local mean arterial pressure (*n* = 15), as is clearly described in the main method section, but due to limited word count, not included in the abstract. As should now be clearer in the abstract, both venous pressure and the estimates of local MAP are of equal size, *n* = 15. (Line 30-31).

**Reviewer 2**

*The authors should add the skin blood flux measurements in the methods section and describe the results accordingly (excluding its reference from the introduction line 66).*

As requested, details of the skin blood flux measurements carried out during pilot testing have been added to the methods section (Line 216-221), and the reference in the Introduction removed.

*Lines 121-127: Please add the author’s hypotheses.*

Thank you for this suggestion, our initial hypotheses have been added to the final section of the Introduction (Lines 135-139)

*Lines 144-146: What is the rationale for this intermittent protocol (i.e., pressure applied and cycle time)? Was it based on previous studies? If so, please provide the references.*

Following an initial transient increase, negative pressure, if sustained, induces a *reduction* in blood flux or blood velocity, often to levels below the pre-intervention baseline (e.g. (Lott et al., 2009; Sundby et al., 2016). For obvious reasons this effect sustained negative pressure is likely to be detrimental to local perfusion if considered from the perspective of being a clinical treatment strategy. Therefore, by applying negative pressure as intermittent cycles, the periods where blood flow is increased during the negative pressure phases can be maximised, and negative pressure only sustained until the point where blood flow is expected to fall below baseline levels.

Previous work (Sundby et al., 2016) has identified cycles of 10-sec negative pressure, 7-sec atmospheric pressure to be the optimal durations to capitalise on the effect of augmenting blood flow, while returning to atmospheric pressure only for the duration required to offset the vasoconstrictive effect of the negative pressure phase, thus ensuring the greatest mean blood flow over a full intermittent negative pressure cycle. As requested, we have included a brief justification for this and the appropriate reference within the Method section (Line 162-163).

Lott, M. E. J., Hogeman, C., Herr, M., Bhagat, M., Kunselman, A., & Sinoway, L. I. (2009). Vasoconstrictor responses in the upper and lower limbs to increases in transmural pressure. *Journal of Applied Physiology*, *106*(1), 302–310. https://doi.org/10.1152/japplphysiol.90449.2008

Sundby, Ø. H., Høiseth, L. Ø., Mathiesen, I., Jørgensen, J. J., Weedon-Fekjaer, H., & Hisdal, J. (2016). Application of intermittent negative pressure on the lower extremity and its effect on macro- and microcirculation in the foot of healthy volunteers. *Physiological Reports*, *4*(17), e12911. https://doi.org/10.14814/phy2.12911

*The data from the Supplementary Figure is important and should be included in the main manuscript.*

Thank you for this point. We agree and so the figure (Fig. 3) detailing the invasive arterial pressure measurements has been returned to the main manuscript.

*Figure 2: It is recommended to add a panel with the group average and individual data of the nadir responses. This will help the readers to better visualize the interindividual variability.*

Thank you for this suggestion, we agree that this is important particularly given the relatively limited sample size. We have included the complete plots for each individual participant in the figure (*n* = 15), with the mean and SD for the group remaining highlighted as previously. We believe this now conveys much richer information about the dataset, in addition to addressing your recommendation.

**Reviewer 3**

*Line 59 to 61 - The authors wrote: Although the systemic effects of LBNP are well documented (3), the mechanisms underlying the positive clinical effect of INP remain unclear, but may relate to oscillations in shear stress within the macro (4) and micro - vasculature (5,6) Since this is the first time the acronym LBPN appears in the text, you should spell it out.*

Thank you for spotting this oversight. This has been added (Line 67).

*Lines 286: The authors wrote: Temporal changes during the during both postures are presented in Figure 1. I think this should be Temporal changes during both postures are presented in Figure 1.*

Again, thank you for spotting the error. This has been corrected (Line 334).

*Line 469 to 472 - The authors wrote: Similarly, the arterial pressure measurements we obtained were sensitive to changes in limb position, and mechanical perturbation (e.g. tapping or flushing) of the artery itself, therefore, we are therefore confident that our local arterial pressure measures would detect any relevant influence of an external pressure change. The word therefore is spelled twice. Please correct into Similarly, the arterial pressure measurements we obtained were sensitive to changes in limb position, and mechanical perturbation (e.g. tapping or flushing) of the artery itself, therefore, we are confident that our local arterial pressure measures would detect any relevant influence of an external pressure change*

Corrected (Line 617). Thank you again.
