# Supplementary material for: External negative pressure transiently reduces intravenous pressure and augments the arteriovenous pressure gradient in the affected limb segment
Source: PLoS One. 2024 Dec 5;19(12):e0315231. doi: 10.1371/journal.pone.0315231 (PMC11620561; doi:10.1371/journal.pone.0315231)
Supplement: Attachment — Submitted filename: Response to reviewers 2.docx [file pone.0315231.s003.docx]

**Response to reviewers 29-10-24 (Second Revision)**

***External negative pressure transiently reduces intravenous pressure and augments the arteriovenous pressure gradient in the affected limb segment.***

Many thanks for the further assistance with improving our manuscript. Responses to any new (second revision) comments are presented below.

**Reviewer 1**

Reviewer appears absent.

**Reviewer 2**

All previous comments addressed.

**Reviewer 3**

Reviewer appears absent.

**Reviewer 4**

*Improvements in transitions, linking the literature review more closely to the study’s aims, and clarifying complex terminology would enhance its accessibility and impact.
The Methods section is thorough, but minor adjustments are needed in participant recruitment, data handling, and statistical analysis to ensure reproducibility.
The Results section presents robust, relevant data, but could be strengthened by improving data presentation clarity, providing more context, and discussing the physiological relevance in greater depth.
The discussion is solid, offering meaningful comparisons with existing literature and highlighting clinical implications, but would benefit from deeper mechanistic insights, more exploration of arterial data, and clearer articulation of clinical relevance.
These enhancements would position the study more effectively within vascular research and increase its potential impact on future clinical applications.*

Thank you for these comments. We have addressed the above recommendations within the Revised Manuscript. Changes are highlighted in Red text and tracked throughout the document. Specifically, we have aimed to improve the flow and links between aims within the paper, as well as mechanistic discussion particularly in relation to the arterial measures.

We stress that given our investigation of a group of healthy volunteers, we have made limited speculation as to the likely mechanisms underpinning any effects among clinical groups or patients with vascular disease. We have however tried to address this as best as possible given the lack of data specific to such patients, while avoiding unfounded speculation, or over-interpretation of our data.

**Reviewer 5**

No specific concerns or comments noted.
